# Improved delivery of the OVA-CD4 peptide to T helper cells by polymeric surface display on *Salmonella*

**DOI:** 10.1186/1475-2859-13-80

**Published:** 2014-06-04

**Authors:** Junjie Zhang, Leon De Masi, Beena John, Wenxin Chen, Dieter M Schifferli

**Affiliations:** 1 State Key Laboratory of Agrobiotechnology and College of Biological Sciences, China Agricultural University, Beijing 100193, People’s Republic of China; 2 Department of Pathobiology, University of Pennsylvania School of Veterinary Medicine, Philadelphia, Pennsylvania 19104, USA; 3 College of Food and Biological Engineering, Zhengzhou University of Light Industry, Zhengzhou, Henan province 450002, People’s Republic of China

**Keywords:** Autotransporter, T5SS, *Salmonella*, MisL, Ovalbumin

## Abstract

**Background:**

Autotransporter proteins represent a treasure trove for molecular engineers who modify Gram-negative bacteria for the export or secretion of foreign proteins across two membrane barriers. A particularly promising direction is the development of autotransporters as antigen display or secretion systems. Immunologists have been using ovalbumin as a reporter antigen for years and have developed sophisticated tools to detect specific T cells that respond to ovalbumin. Although ovalbumin-expressing bacteria are being used to trace T cell responses to colonizing or invading pathogens, current constructs for ovalbumin presentation have not been optimized.

**Results:**

The activation of T helper cells in response to ovalbumin was improved by displaying the OVA-CD4 reporter epitope as a multimer on the surface of *Salmonella* and fused to the autotransporter MisL. Expression was optimized by including tandem *in vivo* promoters and two post-segregational killing systems for plasmid stabilization.

**Conclusions:**

The use of an autotransporter protein to present relevant epitope repeats on the surface of bacteria, combined with additional techniques favoring stable and efficient *in vivo* transcription, optimizes antigen presentation to T cells. The technique of multimeric epitope surface display should also benefit the development of new *Salmonella* or other enterobacterial vaccines.

## Background

*Escherichia coli* is the prototype bacterium used for the production of desired foreign proteins *in vitro*. A particularly interesting approach has been the export of foreign proteins on the bacterial surface of *E. coli* by taking advantage of the specific inherent properties of autotransporter proteins. These proteins, which represent a category of type V secretion systems of Gram-negative bacteria, are exported to the periplasm by the sec machinery and assembled into the outer membrane by the Bam and Tam proteins [1-3]. The carboxy-terminal end of an autotransporter protein forms a beta-barrel structure with a central pore originally thought to channel the amino-terminal side of the protein or passenger domain to the bacterial surface. Newer models propose that Bam participates in making the pore, implying that the term “autotransporter” is a misnomer [3]. For some autotransporter proteins, the translocated segment is cleaved and released from the bacteria, which can be useful for direct purification procedures from spent medium. Some cleaved translocators remain surface associated by non-covalent bond. Whether secreted or on the bacterial surface, translocators can act as adhesins, mediators of biofilm formation, enzymes for intercellular spreading, cytotoxins or modulators of immune responses [3]. Early studies recognized the use of autotransporters as export machineries for foreign proteins and antigens. Translocator domains of a variety of autotransporter proteins have been modified with in-frame fusions of recombinant proteins for display on the bacterial surface or for delivery to the spent medium [4,5]. Among these autotransporters, the *Salmonella* MisL autotransporter adhesin [6] has previously been engineered to express four copies of the Plasmodium falciparum immunodominant epitope (NANP) on the surface of *Salmonella enterica*[7]. MisL was able to express even more copies of NANP on *E. coli* and to release them in the medium after the addition of a cleavage site for the surface protease OmpT [8]*.* The epitopes were shown to induce immune responses. Similarly, viral and parasitic protein epitopes were successfully expressed by recombinant MisL on *Salmonella* vaccine strains and induced epitope-specific immune responses with protective properties [9,10].

Here, we took advantage of the transport property of MisL to export a fused antigenic model peptide on the surface of *Salmonella* as an activation signal for specific cellular immune responses. The generation of a T helper cell 1 (Th1) immune response is crucial for successful control of the facultative intracellular pathogen *Salmonella* and several studies have highlighted the importance of the CD4+ T cell response during infection [11]. The ability to track antigen specific T cells is important for understanding the initiation and maintenance of T cell responses during various infections and in response to vaccines [12]. Since endogenous T cells specific for any given antigen are present in small numbers, it makes the tracking of such cells difficult during the early phases of an immune response before clonal expansion has occurred. Adoptive transfer models using TCR-transgenic T cells specific for model antigens such as ovalbumin have thus provided a vital tool for tracking antigen specific T cell responses [13-15]. A key aspect of such studies is to obtain efficient and stable expression of a foreign antigen by a genetically engineered pathogen. The expression systems in *Salmonella* using full-length ovalbumin constructs available thus far have resulted in suboptimal responses *in vivo*[16-18]. The current investigation highlights the use of a novel construct based on the polymeric surface display of an ovalbumin reporter epitope to amplify the signal for the improved activation and detection of cognate CD4+ T cells.

## Results

### Construction, expression and export of the MisL-OVA-CD4 fusion proteins

**Figure 1:**
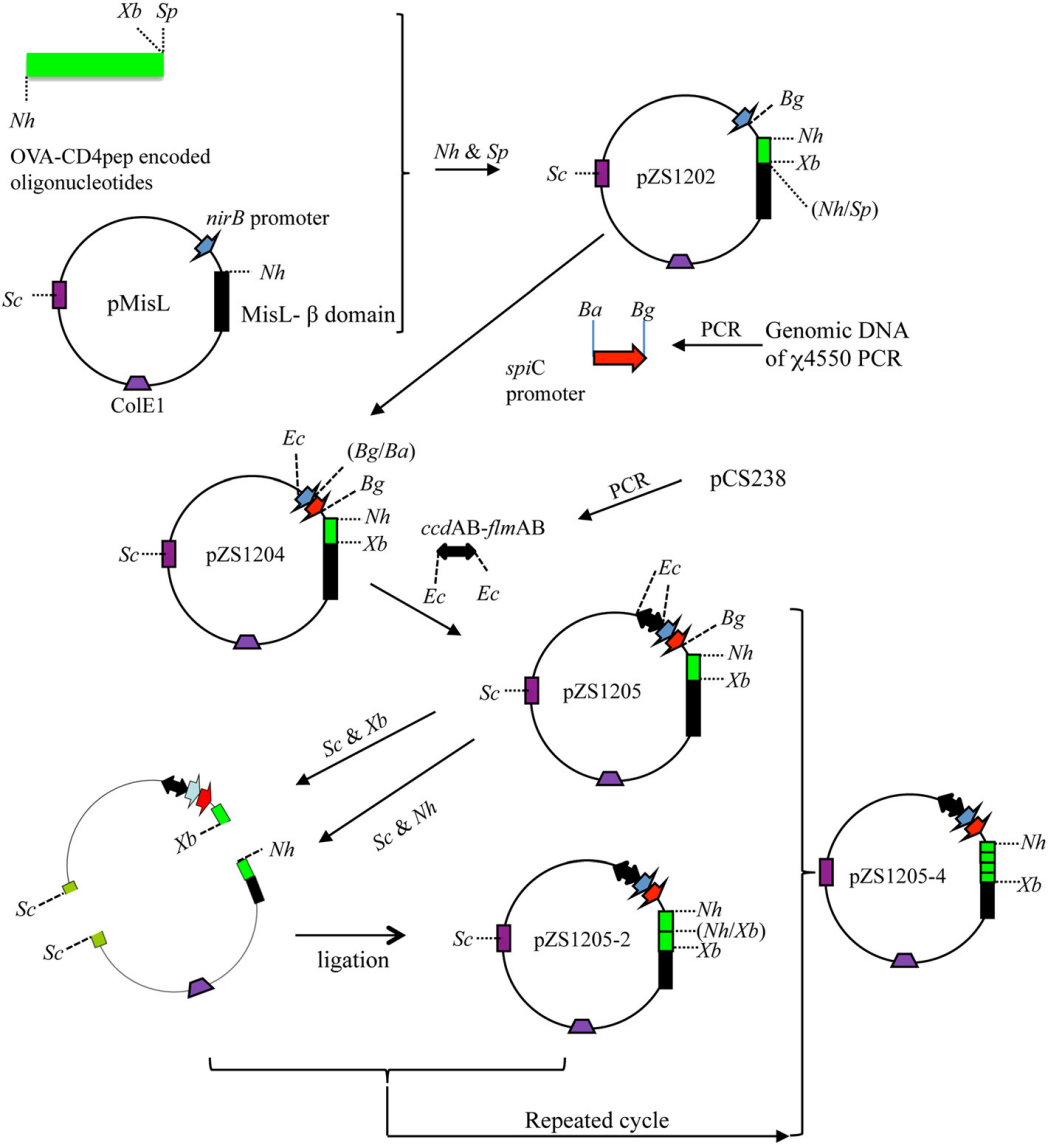
**Plasmid constructions, as described in Methods.**

**Figure 2:**
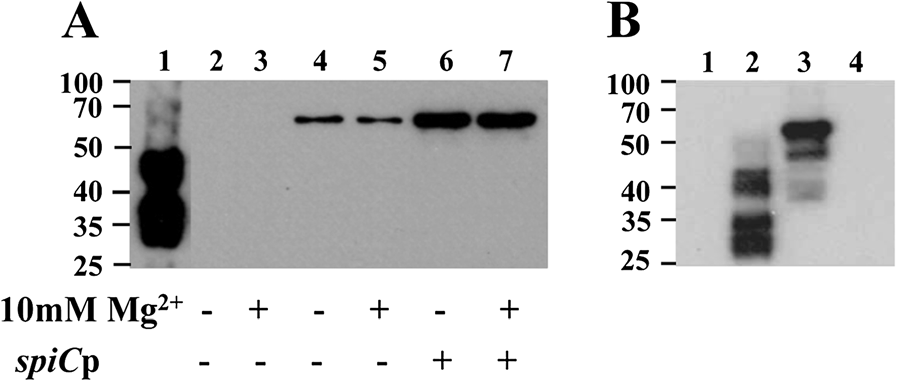
**Expression of OVA-CD4pep-MisL fusion proteins determined by western blot analysis using an anti-OVA-CD4 peptide polyclonal antibody (A) Ovalbumin (lane 1), strain CS4551 with pnirBLTBsp-MisL (lanes 2 and 3), pZS1202 which expresses the (OVA-CD4)-MisL fusion protein from the *nirB *** **promoter (lanes 4 and 5), or pZS1204 expresses the (OVA-CD4)-MisL fusion protein from the *nirB *** **and *spiC *** **promoters in tandem (lanes 6 and 7); (B) Bovine serum albumin (lane 1), ovalbumin (lane 2), untreated (lane 3) or proteinase K-treated (lane 4) outer membrane proteins of *Salmonella *** **strain SL7207 pZ1204.**

The *Salmonella* autotransporter protein MisL was used to display a specific MHC class II ovalbumin peptide (OVA-CD4) on the surface of a *Salmonella* vaccine vector. The OVA-CD4 peptide was fused through genetic engineering to the N-terminus of the translocator domain of MisL. The construct was based on the p*nir*LTB-MisL plasmid [7] that encodes a truncated MisL protein with the signal sequence of the heat-labile enterotoxin of *E. coli* in frame. This truncated MisL lacks the amino-terminal 423 amino acids of the mature MisL. The expressed MisL protein included an additional 210 amino acids of its alpha domain to avoid potential protein misfolding in the periplasm and ensure correct conformation in the outer membrane for transit of the passenger domain to the bacterial surface and display of its N-terminus [7,8]. The plasmid used the *nirB* promoter that is activated under the anaerobic conditions of the intestinal environment and induces improved systemic immune responses towards foreign antigen expressed by orally administered *Salmonella* vaccine strains [19-21]. This vector is not a high copy number plasmid as antibody responses against antigens delivered by *Salmonella* are improved when expressed from low to medium copy number plasmids that minimize metabolic stress upon the carrier strain [22,23]. Previous addition of the *spiC* promoter, which is activated in antigen-presenting cells, was used successfully to express the MisL protein or the 987P fimbriae of *E. coli* as viral epitope carriers on the surface of a *Salmonella* vaccine vector. Oral immunization experiments in mice also suggested improved *in vivo* expression by showing better humoral immune responses against the foreign antigens [10,24]. Thus, we prepared a similar promoter tandem construct by inserting the *spiC* promoter between the *nirB* promoter and the Shine Delgarno site for MisL transcription (Figure 1). Expression of the OVA-CD4-MisL fusion protein was evaluated with *Salmonella* strain SL7207 carrying either pZS1202 (*nirB*p) or pZS1204 (*nirB*p and *spiC*p). Using an antibody specific for the OVA-CD4 epitope, the fusion protein of expected size (approximately 60 kDa) was detected by western blot analysis for both constructs with approximately three times more protein being expressed when both promoters were used, as determined by densitometry (Figure 2A). The OVA-CD4 specific antibody was confirmed to react with ovalbumin but not with *Salmonella-*expressing MisL alone. The detection of the fusion protein in *Salmonella* grown under aerobic conditions suggests some promoter leakiness, although the high affinity of the anti-OVA-CD4 antibody used in these assays might render detection very sensitive. Previous data indicated that magnesium activated fimbrial subunit gene expression from the *spiC* promoter in a *phoP* mutant [24]. However, using the same mutant strain, a significant effect due to magnesium was not detectable for MisL, possibly due to the fact that MisL is a much larger protein than the previously studied fimbrial subunit. In order to demonstrate that the OVA-CD4-MisL fusion protein was crossing the cytoplasmic membrane and behaved like the native MisL protein, outer membranes prepared from strain SL7207 carrying the plasmid with the *misL* fusion gene were analyzed by western blotting. The OVA-CD4-MisL fusion protein was detected in the outer membrane preparation using the ovalbumin-peptide specific antibody (Figure 2B), showing that the fusion protein was not affected in its export and localization properties. Moreover, fused OVA-CD4 peptide was susceptible to proteinase K degradation, characterizing its accessibility on either one or both outer membrane surfaces.

### Stabilized plasmids

**Figure 3:**
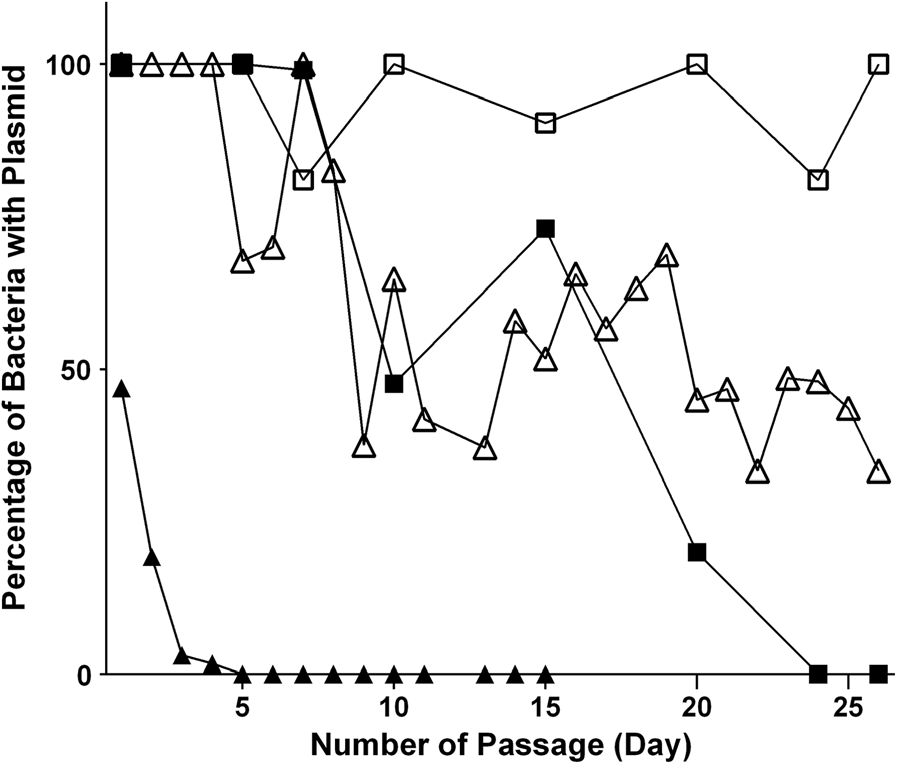
**Plasmid segregational stability.***Salmonella* SL7207 with the stabilized pZS1205 plasmid (☐) or the nonstabilized pZS1204 plasmid (￭), each expressing (OVA-CD4)-MisL, with the stabilized pZS1205-4 (△) that expresses four copies of OVA-CD4 fused to MisL, or pUC18-OVA (▲) that expresses full-length ovalbumin were grown in LB broth at 37°C without antibiotics. Serial passages were conducted daily by diluting the overnight cultures into fresh media (10^-3^ dilutions for the first week and 10^-5^ dilutions for later). Plasmid stability was estimated by calculating the numbers of ampicillin-resistant CFUs divided by the total number of CFUs and multiplying the number by 100 to obtain a percentage of bacteria containing the plasmid.

To ensure that attenuated *Salmonella* strains would keep the OVA-CD4-MisL plasmid construct in the absence of antibiotic selection after administration to mammalian hosts, two post-segregational killing systems from the F plasmid of *E. coli*, the *fimAB* and *ccdAB* genes were engineered into the plasmid (Figure 1). Efficiency of plasmid maintenance was investigated by growing *Salmonella* SL7207 with or without the stabilized plasmid in antibiotic-free media for approximately 530 generations. The plasmid-bearing rate was calculated by dividing the number of bacteria recovered from antibiotic plates by the number of bacteria recovered from antibiotic-free plates. As shown in Figure 3, plasmid pZS1204 with no stabilization system was gradually lost during after 20 daily passages, however plasmid pZS1205 was kept in essentially all the bacteria. A derivative of the latter plasmid, pZS1205-4, which expresses four copies of the OVA-CD4 was 100% maintained for one week and in nearly half of the bacteria for 26 days. In contrast, the high copy number plasmid pUC18-OVA that constitutively expressed the full-length ovalbumin protein was lost in five days. These results confirmed that high copy number plasmid constructs have the undesirable effect of being unstable in the absence of antibiotic selection. More importantly, the data demonstrated that the *fimAB* and *ccdAB* genes have an efficient plasmid stabilization property that prolongs the life span of foreign antigen-expressing *Salmonella*.

### Surface exposure of the OVA-CD4 epitope peptide on *Salmonella*

**Figure 4:**
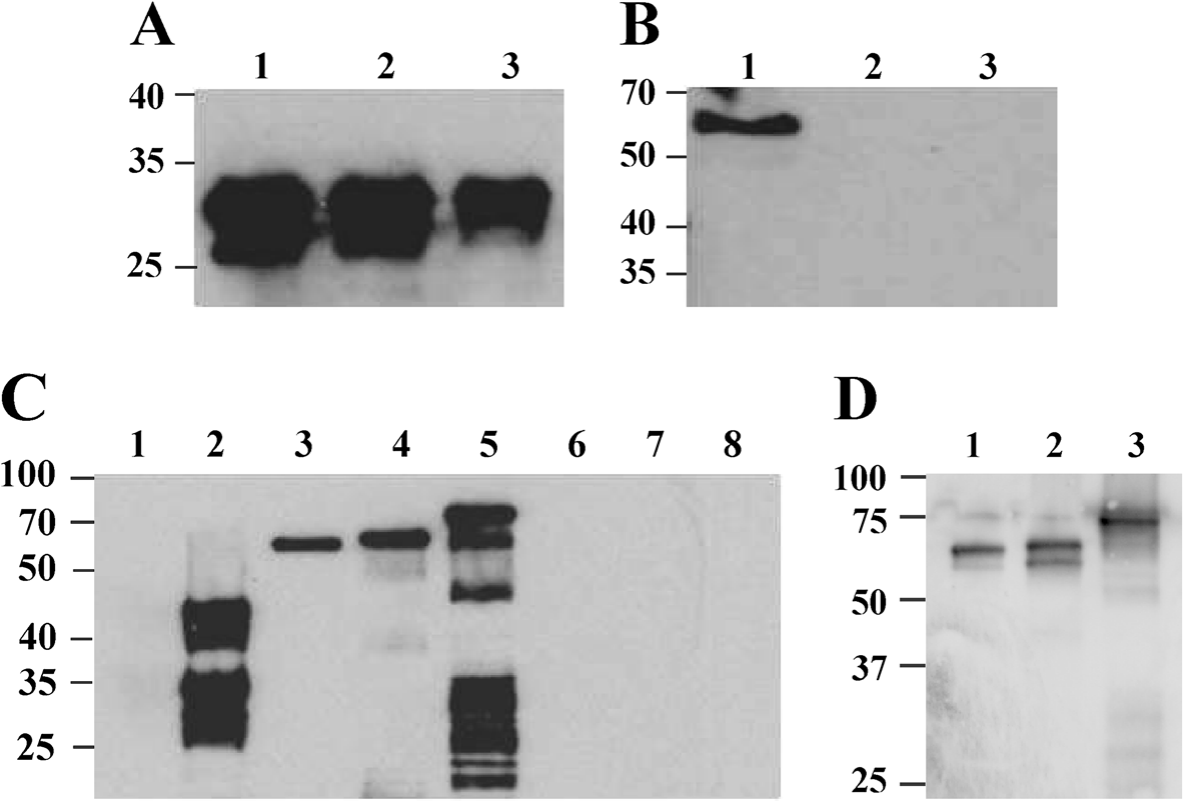
**Surface exposure of (OVA-CD4)-MisL fusion protein detected by western blot analysis.***Salmonella* SL7207 pZS1205 treated with 0 (lane 1), 11 (lane 2) and 33 (lane 3) μg/ml proteinase K and probed with **(A)** anti-β-lactamase pAb or **(B)** anti-OVA-CD4 peptide pAb antibodies, respectively. **(C)***Salmonella* SL7207 expressing MisL fusion proteins with one (pZS1205, lanes 1 and 6), two (pZS1205-2, lanes 4 and 7) or four (pZS1205-4, lanes 5 and 8) copies of the OVA-CD4 epitope; untreated (lanes 3 to 5) or treated (lanes 6 to 8) with proteinase K. Bovine albumin (lane 1) and ovalbumin (lane 2), as negative and positive antibody controls. **(D)***Salmonella* SL7207 *pgtE* expressing MisL fusion proteins with one (pZS1205, lane 1), two (pZS1205-2, lane 2) or four (pZS1205-4, lane 3) copies of the OVA-CD4 epitope.

To determine whether the OVA-CD4 portion of the fusion protein was located on the periplasmic or extracellular face of the outer membrane, intact *Salmonella* expressing the fusion protein were subjected to treatments with various concentrations of proteinase K. Presence of the OVA-CD4 peptide was then determined by western blot analysis with the OVA-CD4 specific antibodies (Figure 4A). Parallel western blots were probed for the periplasmic β-lactamase with an anti-ampicillinase antibody to evaluate concentrations of proteinase K that would be effective on the bacterial surface without permeating into the periplasmic compartment (Figure 4B). Proteinase K at a concentration of 11 μg/ml completely degraded the OVA-CD4 peptide without affecting the periplasmic β-lactamase, indicating that the latter peptide was exposed on the bacterial surface. A comparable result was obtained with higher concentrations of proteinase K, albeit a partial proteolytic effect on the β-lactamase suggested some permeation of the protease.

### Construction and display of oligomeric OVA-CD4 epitopes on the surface of *Salmonella*

**Figure 5:**
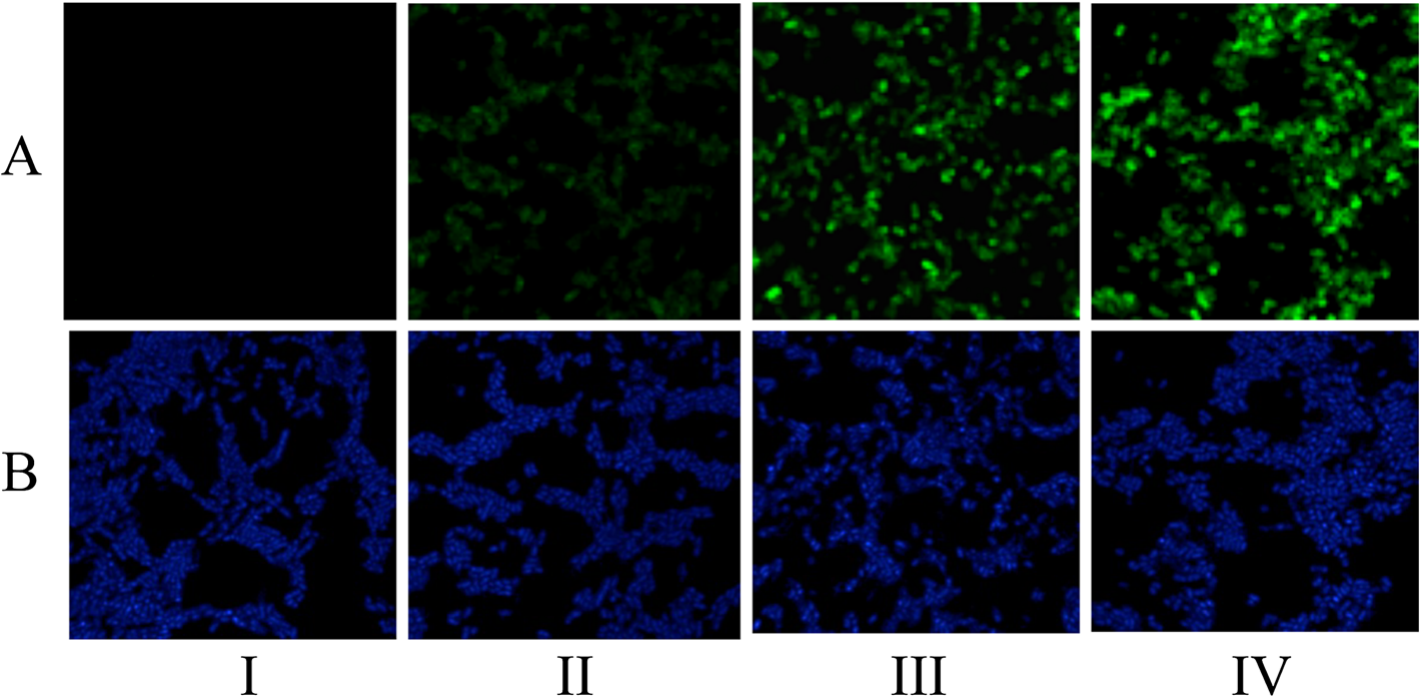
**Confocal microscopy of (A) *Salmonella *SL7207 labeled with rabbit anti-OVA-CD4 antibodies and FITC-conjugated anti-rabbit antibodies, and (B) DAPI-stained *Salmonella *SL7207.***Salmonella* SL7207 carried p*nirB*LTBsp-MisL (I), pZS1205 (II), pZS1205-2 (III) or pZS1205-4 (IV).

Having engineered OVA-CD4 encoded DNA to be flanked by restriction sites with matching 5′ extensions (XbaI and NheI) on plasmid pZS1205, it was possible to construct new homocomposite plasmids that add OVA-CD4 peptides in tandem to the amino-terminal end of MisL. We prepared plasmids capable of expressing MisL fusion proteins carrying dimeric and tetrameric OVA-CD4 epitopes and compared their levels of expression and surface display. Western blot analysis of these constructs showed that each addition of OVA-CD4 epitope copies to MisL significantly increased expression of the fusion protein (Figure 4C). Densitometry of the steady-state detectable proteins indicated that the (OVA-CD4)_2_-MisL dimer and (OVA-CD4)_4_-MisL tetramer constructs had approximately 2.7 and 4.8 times more OVA-CD4 epitopes, respectively, than the (OVA-CD4)_1_-MisL monomeric epitope fusion protein. We speculated that the lower molecular bands seen with the OVA tetramer were due to degradation of surface epitopes by the outer membrane protease PgtE [24], therefore we repeated the experiment with an insertion mutant of *Salmonella* SL7207 deficient for PgtE expression. Densitometry revealed greatly reduced degradation products for the tetramer construct and 1.7 and 4.4 times more OVA-CD4 epitopes were expressed with the dimer and tetramer MisL fusion proteins compared to the monomer construct (Figure 4D). All the ovalbumin epitopes expressed by *Salmonella* were accessible to the proteinase K proteolytic activity, consistent with their surface-exposure. The increased numbers of smaller products for the tetramer construct in the absence of proteinase K pointed towards bacterial auto-degradation processes. Surface display of the OVA-CD4 epitope was visually confirmed by fluorescence microscopy. Using the OVA-CD4 specific antibody with a FITC-labeled secondary antibody to label the surface of *Salmonella* SL7207 expressing one, two or four copies of OVA-CD4 fused to MisL, the intensity of the fluorescence increased accordingly, as determined by the number of additional epitope copies (Figure 5). Fluorescence intensities per bacterial cells using the NIH ImageJ software suggested that the OVA-CD4 dimer and tetramer fusion constructs displayed respectively 2.1 and 3.4 times more epitope as compared to the monomer construct. Fluorescence intensities with the PgtE mutant containing the same plasmids showed similar increases in epitope expression, with 2.4 and 5 times more epitope displayed by the dimer and tetramer constructs respectively (data not shown). The incremental levels of fluorescence and western blot bands confirmed higher expression and surface exposure of the OVA-CD4 peptide with increased numbers of tandem repeats in the constructs.

### Expression of full-length ovalbumin versus (OVA-CD4)_4_-MisL

**Figure 6:**
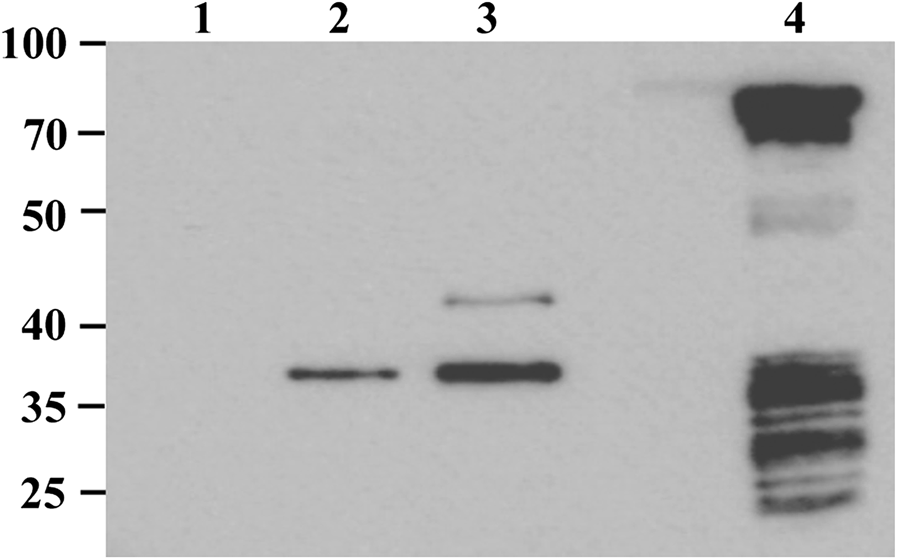
**Expression of the OVA-CD4 peptide.***E. coli* JM109 pUC18 (lane 1), with pUC18-OVA uninduced (lane 2) or IPTG-induced (lane 3) or with pZS1205-4 (lane 4).

The relative efficiency of OVA-CD4 epitope expression harboring pZS1205-4, which encodes the peptide tetramer fused to MisL, or pUC18-OVA, which encodes the full-length protein [25], was compared by western blot analysis with the anti-OVA-CD4 antibody (Figure 6). *E. coli* was used as host to compare ovalbumin expression under uninduced and induced conditions. The signal of the MisL fusion construct was 2.6 or 5.6 times stronger than the one from the full-length protein with or without IPTG-induction, respectively.

### *In vitro* antigen presentation

**Figure 7:**
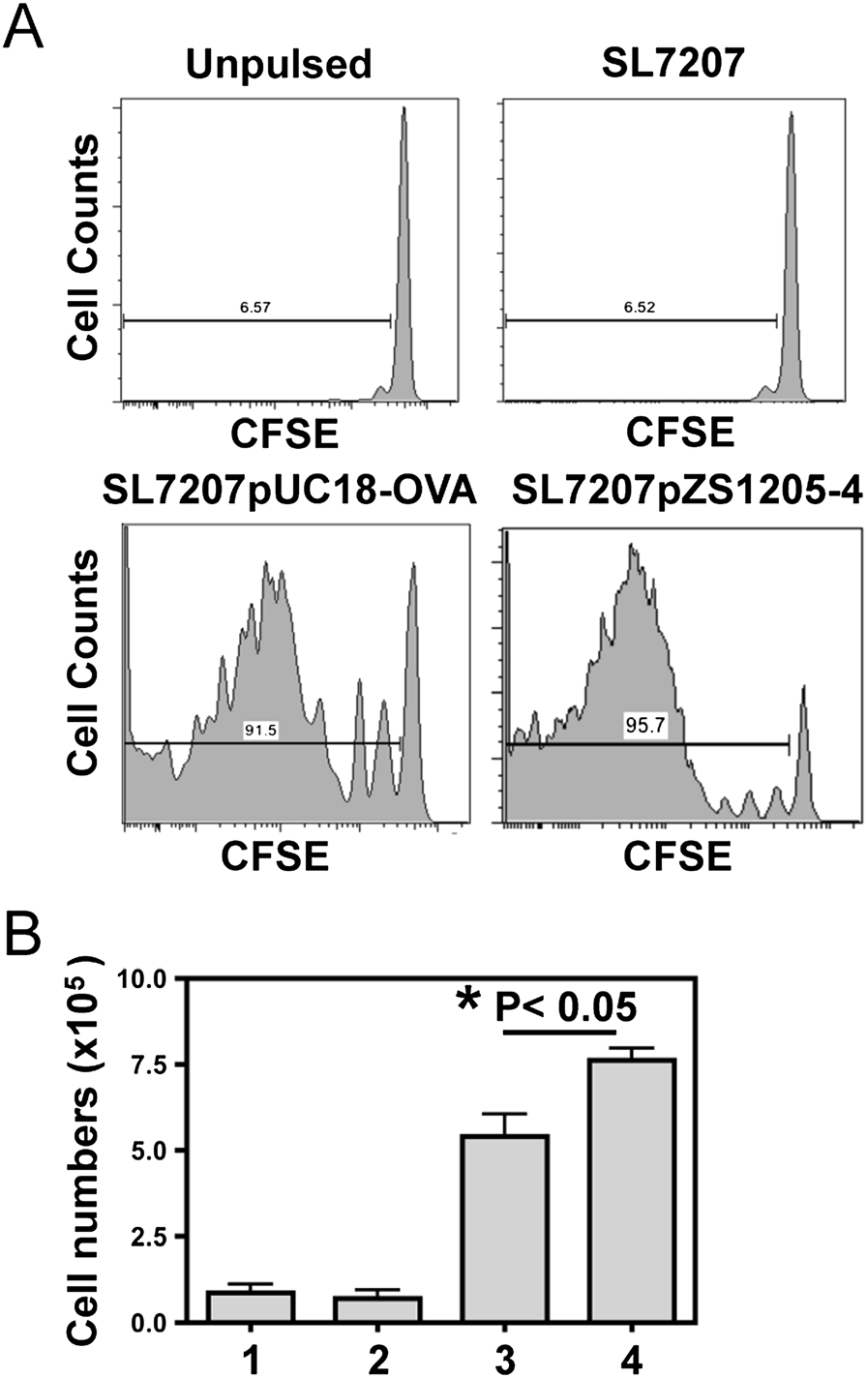
***In vitro *antigen presentation. (A)** The CFSE dilution profile of OTII CD4+ T cells which were co-cultured with APCs that were either (1) left unpulsed (PBS) or pulsed with *Salmonella* (2) SL7207,(3) SL7207pUC18-OVA or (4) SL7207pZS1205-4 [(OVA-CD4)x4]. The cells were gated on CD3+ CD4+ CD45.1+ fraction of the live lymphocytes. **(B)** The total numbers of OTII T cells recovered from the four co-cultures (as labeled above) at the end of the four-day cultures is shown. The data are representative of three independent experiments.

The efficacy of the *Salmonella* SL7207pZS1205-4 [(OVA-CD4)_4_-MisL] constructs in stimulating ovalbumin specific CD4 T cell responses was tested using *in vitro* antigen presentation assays with OTII TCR transgenic system. The responses obtained with the current construct were compared to a known full-length ovalbumin expressing system (pUC18-OVA) that has been used extensively in the field [25,26], using the same *Salmonella* strain. The OTII T cells were labeled with CFSE in these *in vitro* assays and the dilution of CFSE was used to measure T cell proliferation. When T cells were co-cultured with APCs pulsed with parental strains of *Salmonella* that do not express ovalbumin epitopes, they did not undergo proliferation as evident from the lack of CFSE dilution (Figure 7A). APCs pulsed with *Salmonella* expressing the full length ovalbumin or (OVA-CD4)_4_-MisL resulted in activation of T cells and their proliferation. The OTII T cell expansion was significantly higher in response to (OVA-CD4)_4_-MisL as compared to full-length ovalbumin, when each was expressed by the same *Salmonella* strain (Figure 7A and B).

### *In vivo* antigen presentation

**Figure 8:**
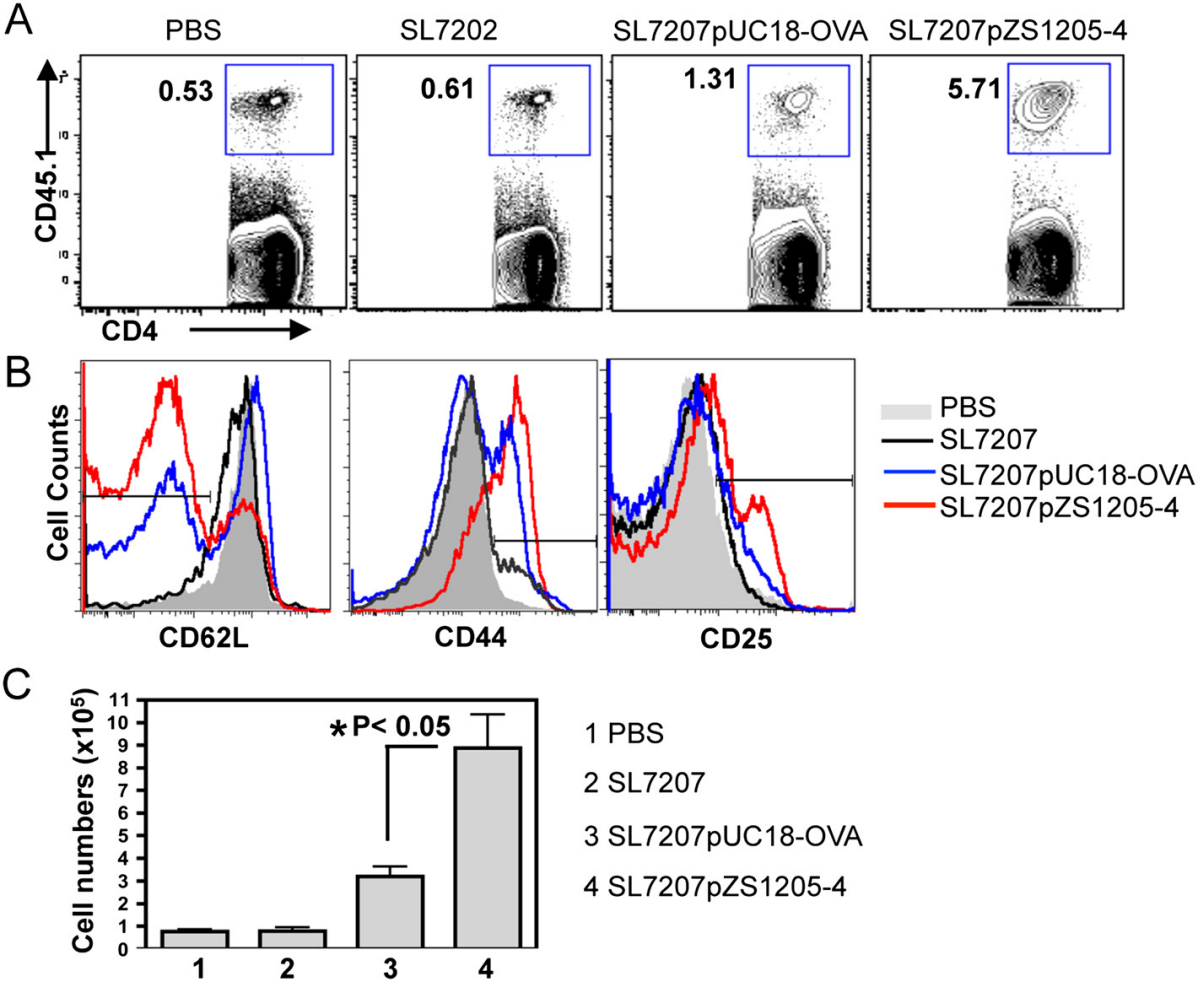
***In vivo *antigen presentation. (A)** The frequency of OTII CD4+ T cells isolated from the mesenteric lymph nodes of mice that were administered orally (1) PBS, (2) *Salmonella* SL7207, (3) SL7207pUC18-OVA or (4) SL7207pZS1205-4 [(OVA-CD4)x4]. The cells were gated on CD4+ CD3+ live lymphocytes and the transferred T cells were identified using the congenic marker CD45.1. **(B)** The expression of the surface markers CD62L, CD44 and CD25 on the gated OTII T cells isolated from the four groups described above; PBS (grey area), *Salmonella* SL7207 (black line), SL7207pUC18-OVA (blue line) and SL7207pZS1205-4 [(OVA-CD4)x4] (red line). **(C)** The total numbers of OTII T cells recovered from the mesenteric lymph nodes of the four groups of mice described above. The data shown are representative of three independent experiments.

To test the efficacy of the various bacterial constructs *in vivo*, an adoptive transfer model was used, as described in the Methods section. The OTII T cells expressed the congenic marker CD45.1 and could thus be tracked in the recipient mice (CD45.2). Seven days after oral infection with the different bacterial strains, the mice were euthanized and lymphocytes were isolated from the spleen and mesenteric lymph nodes and analyzed by flow cytometry. These studies showed that there was an increase in the frequency of the OTII T cells during infection with the ovalbumin-expressing *Salmonella* SL7207 compared to the controls that received PBS or *Salmonella* alone (Figure 8A). The frequency of OTII T cells (CD45.1 CD4+) in the mice that were administered the strain expressing (OVA-CD4)_4_ was higher as compared to the mice infected with the strain that expressed full-length ovalbumin. No deletion of OVA-CD4 repeats was detected during the time of the *in vivo* experiment (data not shown). Based on the analysis of surface marker expression for activation, such as up regulation of CD44 and CD25, and down regulation of CD62L, there was a higher frequency of activated OTII T cells in the mice infected with the (OVA-CD4)_4_-expressing *Salmonella* (Figure 8B). These differences were also apparent in the total numbers of OTII T cells in the mesenteric lymph nodes (Figure 8C) and spleens (data not shown), with significantly higher numbers of OTII T cells present in the mice infected with the (OVA-CD4)_4_-expressing *Salmonella* compared to the mice infected with the full-length ovalbumin-expressing *Salmonella*. Together these data showed that the newly developed construct permits robust expression of the model ovalbumin epitope both *in vitro* and *in vivo* and can be used as reliable model to track antigen specific T cell responses.

## Discussion

Our studies demonstrated that the MisL autotransporter protein was capable of displaying the OVA-CD4 epitope on the surface of *Salmonella* when the epitope was grafted onto the amino-terminal side of MisL. Moreover, MisL fusion proteins engineered to carry the epitope in tandem copies increased the efficiency of surface display. Based on our previous work on foreign antigen expression by *Salmonella*, we designed a prototype plasmid to optimize *in vivo* antigen presentation. For this, a tandem *in vivo* inducible promoters was inserted upstream of the OVA-CD4-MisL open reading frame in a low to medium copy number plasmid and two sets of stabilization systems were introduced into the construct. When compared to a traditional *Salmonella* construct expressing full-length ovalbumin in the cytoplasm from a multicopy number plasmid with a strong constitutive promoter, our prototype construct expressing four OVA-CD4 epitopes was significantly more efficient at presentation for activating CD4 T cells, both *in vitro* and *in vivo*.

A variety of microbes [14,27,28], including wild type or attenuated entero-invasive *Salmonella*[16,25,29], have been genetically modified to express ovalbumin with its well-characterized CD4 and CD8 T cell epitopes as a reporter system to study cellular immune responses in hosts. Many of these studies did not attempt to optimize the expression level and bacterial delivery mechanism for the most efficient immune recognition of the relevant epitopes. For this study, we designed and tested an improved ovalbumin reporter construct based on previous studies with *Salmonella* vaccine vectors that have established general principles for the improvement of immune responses. First, surface display of a foreign epitope was reported to induce a better immune response than when the same epitope remained in the bacterial cytoplasm [20]. A recent study suggested that location on the bacterial surface was even more important for immunity than high abundance or immunodominance since *Salmonella* mainly survive in antigen-presenting cells, rendering surface antigens more accessible to the cell for processing and presentation [30]. Second, surface-exposed foreign epitopes presented as multimers induced better antibody responses than the same epitopes presented as monomers on a Salmonella vaccine vector [10]. Because it is capable of expressing antigens on a *Salmonella* surface, the MisL protein has the advantage of tolerating large fragments of proteins in a fusion construct. This property was used to incrementally add OVA-CD4 epitopes to MisL and demonstrated a corresponding stepwise increase of epitope expression on the surface of *Salmonella*. The addition of tandem epitope copies did not affect bacterial growth. Although surface expression of larger amounts of foreign epitopes can induce some degradation by the PgtE protease of *Salmonella*, as shown previously [24] and here with four copies of the OVA-CD4 epitope, PgtE did not significantly affect steady-state expression of the surface exposed epitope. A third generic rule to improve antigen delivery for better immune responses is the avoidance of high copy number plasmids that express large foreign antigen from constitutive promoters [23,31]. Such vector constructs are metabolic stressors for the bacteria and are rapidly eliminated or mutated, resulting in reduced levels of immune response [22]. In general, metabolic stress can be minimized by both the use of a low copy number plasmid and the expression of the smallest protein fragment needed for an experimental task, such as a major immunogenic epitope fused to a *Salmonella* protein. Here, we demonstrated that the multicopy number plasmid pUC18-OVA that expresses the full-length ovalbumin was lost *in vitro* after only a few passages in the absence of antibiotic selection, suggesting that it is not an optimal construct. In contrast, the low copy number pZS1204 was far more stable than pUC18-OVA. Safe remedies to stability issues for the expression of foreign proteins have been the use of chromosomal integration or single-copy number plasmids together with strong constitutive or *in vivo* inducible promoters [32-34]. As an alternative to stabilize protein expression, one can borrow plasmid post-segregational killing genes. We used the latter approach by inserting a cassette comprising two of these set of genes that we have previously used with success [10] to obtain pZS1205 and confirmed significantly improved plasmid stability over 500 generations. In addition, approaches shown to augment the expression of ovalbumin or other foreign antigens in animal models were based on the engineering of *in vivo*-activated promoters [35-37]. For this study, we took advantage of a tandem promoter system that we had previously shown to express antigen efficiently both *in vitro* and *in vivo*[10,24].

The improved efficiency of antigen presentation with the engineered *Salmonella* displaying stably several copies of the OVA-CD4 epitope on its surface was demonstrated here by the enhanced CD4+ T cell multiplication both *in vitro* and *in vivo*, using a classical bacterial full-length ovalbumin construct in the same bacterial background for comparison. Although we did not evaluate the individual effect of each construction step on this immune response, we used a systematic approach based on an extensive body of literature and our previous studies to engineer an efficient ovalbumin reporter system. Among the many autotransporter proteins that are capable of presenting heterologous surface antigens or secreted proteins, MisL has already demonstrated its adaptability as an antigen delivery system in the context of experimental *Salmonella* vaccines [7-10,38]. Some of these studies showed that MisL was tolerant to short epitope multimers. Here, we confirmed multimer display with a longer epitope fused to MisL and demonstrated efficient activation of CD4+ T cells. The identification of an increasingly larger panoply of autotransporter proteins capable of exporting foreign proteins might help to partially bypass display or secretion limitations due to the nature, size and number of epitopes added [4,39]. Although the construct described here was developed to create an improved ovalbumin reporter system for the study of CD4+ T cell responses to *Salmonella*, the concept of epitope presentation in the form of tandemly repeated homopolymers might be applicable for other purposes, including their cytoplasmic delivery by a bacterial injection apparatus or invasive mechanism for a CD8+ T response [10,40,41]. Polymerization of protective epitopes should also benefit vaccine development.

## Methods

### Bacterial strains, media and reagents

The bacterial strains used in this study were as follows: *E. coli* DH5 alpha (F^-^φ80*lacZ*ΔM15Δ(*lacZYA*-*argF*)U169 *recA*1 *endA*1 *hsdR*17(rk^-^, mk^+^) *phoA supE*44 *thi*-1 *gyrA*96 *relA*1 λ^-^) [42], JM109 [43], *Salmonella enterica* serovar Typhimurium SL7207 (*hisG*46, DEL407 [*aroA*544::Tn10 (Tc^s^)])(derivative of strain SL3261, kind gift from Dr. Bruce Stocker, Stanford University) [44], *S.* Typhimurium χ4550 (*gyrA*1816Δ*asdA*1 Δ(*zhf*-4::Tn*10*) Δ*crp*-1Δ*cya*-1) [45], *S.* Typhimurium CS4551 (χ4550 *phoP::cat*) and *S.* Typhimurium CS4552 (χ4550 *pgtE::aphA*) [24]. The SL7207 *pgtE* mutant was engineered by generalized transduction, using phage P22. Successful transfer was confirmed by PCR. All the strains were grown in Luria-Bertani (LB, Lennox) medium (Difco, Detroit, MI, USA), with the addition of 50 μg/ml DL-alpha, ϵ-diaminopimelic acid (DAP; Sigma, St. Louis, Mo.) for *S.* Typhimurium strains χ4550 and CS4551. Media were supplemented with ampicillin (100 μg/ml), chloramphenicol (30 μg/ml), kanamycin (50 μg/ml), X-Gal (35 μg/ml), and IPTG (0.5 mM), or MgCl_2_ overnight [24] when needed or indicated. Restriction and modification enzymes were from New England Biolabs Inc. (Beverly, MA, USA). Unless specified otherwise, reagents were purchased from Sigma.

**Table 1:** List of plasmids

**Plasmids**	**Characteristics**	**Resistance**	**References**
pGEM®-T		Ap^r^	Promega, Madison, WI
pUC18-OVA	1.2 kb ovalbumin gene in pUC18	Ap^r^	[25]
pZS1201	pGEM®-T Easy Vector - OVA-CD4	Ap^r^	This study
pnirBLTBsp-MisL	pnirB for LTsp-MisL, *colE1*-like *ori*	Ap^r^	[7]
pZS1202	pnirBLTBsp-MisL-OVA-CD4	Ap^r^	This study
pZS1203	pGEM®-T Easy Vector -*spiC*p	Ap^r^	This study
pZS1204	pZS1202 with *spiC*p	Ap^r^	This study
pCS238	pACYC184 with *ccdAB*-*flmAB*	Cm^r^	[10]
pZS1205	pZS1204 with *ccdAB*-*flmAB*	Ap^r^	This study
pZS1205-2	pZS1205 with [OVA-CD4]x2	Ap^r^	This study
pZS1205-4	pZS1205 with [OVA-CD4]x4	Ap^r^	This study

### Plasmid constructs

Table 1 lists all the plasmids used in this study. Plasmids carrying the genes for MisL-[OVA-CD4]_n_ (n = 1, 2 or 4) fusion proteins were engineered to be under the control of two *in vivo*-inducible promoters in tandem (Figure 1) [24]. For this, both strands for the ovalbumin derived CD4 epitope (OVA-CD4: “ISQAVHAAHAEINEAGR”) were synthesized as follows: Forward strand: 5′-**GCTAGC**GGTGGCATTAGCCAGGCCGTGCATGCGGCCCATGCGGAAATTAACGAAGCCGGCCGCGGTGGC**TCTAGAACTAGT**A-3′; Reverse strand: 5′-**ACTAGTTCTAGA**GCCACCGCGGCCGGCTTCGTTAATTTCCGCATGGGCCGCATGCACGGCCTGGCTAATGCCACC**GCTAGC**A-3′. Both strands had an *Nhe*I restriction site at one end, and *Xba*I and *Spe*I restriction sites at the other end. The 5′ ends were phosphorylated and the 3′ end had an additional A for TA cloning. The hybridized strands were ligated into the pGEM®T easy vector (Promega), resulting in plasmid pZS1201. The OVA-CD4 epitope containing fragment from *Nhe*I and *Spe*I, restricted pZS1201 was purified by agarose gel electrophoresis and ligated into the *NheI* restriction site of pnirBLTBsp-MisL [7], resulting in plasmid pZS1202. The *spi*C promoter from strain χ4550 was amplified by PCR, using upper primer 5′- ATGC**GGATCC**AATGCTTCCCTCCAGTTGCCTGTT-3′ and lower primer 5′-ATGCGG**AGATCT**AAATGGGAGTTTCTATCAAATTC-3′, carrying a BamHI or BglII near their 5′ end, [24]. The amplicon ligated into pGEM®T was designated pZS1203. The *Bam*HI *Bgl*II fragment of pZS1203 carrying the *spiC* promoter was ligated into the *Bgl*II site of pZS1202, which is downstream of the nirB promoter sequence [21], resulting in pZS1204, as plasmid with tandem promoters. Two post-segregational killing systems (*ccd*AB and *flm*AB) were amplified by PCR using pCS238 as template and primers 5′-ATCGT**GAATTC**CTGCAGACTGGCTGTGTATAAC-3′ and 5′-ATCGT**GAATTC**CCTGGCAGTCTGGTTGTTCAT-3′[10]. The amplicon was restricted with *Eco*RI and inserted into the corresponding site of pZS1204, creating plasmid pZS1205. A second OVA-CD4 epitope was added to the latter plasmid by restricting pZS1205 with *Nhe*I and *Sca*I or with *Xba*I and *Sca*I, and the two fragments containing the DNA encoding for the OVA-CD4 epitope were purified by agarose gel electrophoresis and ligated to create pZS1205-2. This procedure was repeated to obtain pZS1205-4, which carries 4 tandem copies of OVA-CD4 DNA as an in-frame fusion to MisL. All the amplified and cloned PCR products were checked for sequence accuracy.

### Preparation of outer membrane proteins

Outer membrane fractions were prepared from spheroplasts, as described previously [46]. Bacteria from 10 ml overnight cultures (approximately 10^10^ CFU) were pelleted by centrifugation and suspended in 90 μl of 30 mM Tris–HCl (pH 8.0) with 20% sucrose. 10 μl of lysozyme (1 mg/ml in 0.1 M EDTA) was added and the bacteria were incubated for 30 min on ice. The obtained spheroplasts were stabilized with MgCl_2_ (20 μM final concentration) and centrifuged (16,000 × g, 2 min). The periplasmic proteins were removed with the supernatant and the spheroplasts were resuspended in 100 μl of a 10 mM Tris–HCl (pH8.0), 100 mM NaCl, 10 mM MgCl_2_ solution containing 1 μg of DNase per ml, and lysed by sonication (two times 1 min with a Cup Horn accessory at amplitude output 10, using a model XL2020 sonicator; Heat System, Farmingdale, N.Y.). Cytoplasmic membrane proteins were solubilized by incubating the membranes for 20 min at room temperature with N-lauroylsarcosine, sodium salt (ICN Biochemicals, Cleveland, Ohio) at a final concentration of 0.5% [47]. Residual intact cells were removed by centrifugation at 1,200 × g for 10 min at 4°C. The non-solubilized outer membranes were pelleted by high-speed centrifugation in a Beckman JA-18.1 rotor at 17,000 rpm for 3.0 h at 4°C. The outer membrane pellet was suspended in 200 μl PBS and mixed 1:1 with 2× sodium dodecyl sulfate-polyacrylamide gel electrophoresis (SDS-PAGE) buffer (sample buffer).

### Protease accessibility to surface exposed domains of MisL fusion proteins

Bacteria grown to log phase (A_600_ ~ 0.6) were washed in PBS three times, then divided in four samples. Proteinase K (recombinant, Roche Diagnostics GmbH, Mannheim, Germany) was added to final concentrations of 11, 33 or 100 μg/ml, leaving one sample as negative control. The samples were incubated at 37°C for 30 min, and protease activity was stopped by adding AEBSF [4-(2-Aminoethyl) benzenesulfonyl fluoride hydrochloride] to each sample at final concentration of 0.5 mM. The samples were directly mixed with 2× sample buffer, heated for 10 min at 100°C, and 5 μl per lane were analyzed by SDS-PAGE and western blotting.

### SDS-PAGE and western blotting

For SDS-PAGE and western blotting, bacteria grown to log-phase (A_600_ ~ 0.6) were pelleted (except for the experiments with isolated outer membranes or using proteinase K, as described above), solubilized in sample buffer and boiled for 5 min. Proteins were separated by SDS-PAGE. The gels were analyzed by western blotting, using affinity purified rabbit anti-OVA-CD4 epitope (ISQAVHAAHAEINEAGR) polyclonal antibodies (OVA 323–339, Innovagen, Lund, Sweden) diluted 1:1000 in PBS-0.1% tween 20 (PBS-T) or with rabbit anti-ampicillinase antibodies (5 prime to 3 prime, Inc. Boulder, CO), followed by horseradish peroxidase (HRP)-conjugated secondary antibodies diluted 1:8000 in PBS-T and enhanced chemiluminescence (ECL) for detection, as described previously [46]. Relative amounts of expressed fusion proteins were evaluated by densitometry, using NIH ImageJ software version 1.47n (http://rsb.info.nih.gov/ij/).

### Indirect immunofluorescence assay (IFA)

For IFA, the bacterial strains were spread on glass slides, fixed with cold methanol and incubated with rabbit anti-ovalbumin antibody. The slides were washed three times with PBS-T, incubated with FITC conjugated goat anti-rabbit-IgG and washed again with PBS-T. Dried slides were mounted with 10 μl antifade reagent with DAPI, overlaid with glass coverslip, and images were acquired on a Leica TCS SP5 inverted confocal microscope with a 63× (1.2 NA) water immersion lens. Antibody staining was visualized with 488 nm excitation from an Argon laser and DAPI was excited with a 405 nm pulsed diode laser. Relative fluorescence intensities were calculated from measurements with the NIH ImageJ software.

### Plasmid stability

To determine the persistence of a plasmid in dividing cells, bacteria were passaged in LB broth without antibiotics for 26 days (approximately 530 generations) using daily 10^-3^ dilutions of overnight cultures for the first week and 10^-5^ dilutions for the remainder. Plasmid stability was estimated by determining at several passage points the numbers of antibiotic-resistant live bacteria containing the plasmid (colony-forming units or CFUs on ampicillin-containing LB agar plates) divided by the total number of live bacteria (CFUs on LB agar plates). This number was multiplied by 100 to obtain a percentage of bacteria containing the plasmid [10].

### Mice

OTII TCR transgenic mice on a CD45.1 background were maintained in a specific pathogen-free (SPF) facility in the Department of Pathobiology at the University of Pennsylvania in conformance with institutional guidelines for animal care. C57BL/6 mice were purchased from Taconic farms and housed within the SPF facility. All animal studies were carried out in compliance with the guidelines of the Institutional Animal Care and Use Committee of the University of Pennsylvania.

### OTII T cell isolation

CD4+ T cells were enriched from lymphocytes isolated from the spleen and pooled peripheral lymph nodes of OTII TCR transgenic mice (Ovalbumin specific CD4+ TCR transgenic mice) using CD4+ MACs beads (Miltenyii Biotech). The cells were labeled with the cytoplasmic dye CFSE (carboxyfluorescein diacetate succinimidyl ester, Molecular Probes, Eugene, OR), to track proliferation and used for the *in vitro* assays or transferred into mice for testing *in vivo* efficacy of the bacterial constructs.

### *In vitro* antigen presentation assay

Antigen-presenting cells were isolated from spleens of WT mice; CD11C + cells were enriched from RBC lysed splenocytes using CD11c + MACs beads (Miltenyii Biotech). The purified APCs were resuspended in complete RPMI (10% FBS) and plated in 24 well plates (1×10^5^ cells/well). The APCs were pulsed with the various bacterial constructs at a multiplicity of infection (MOI) of 10 for 2 hours, following which the cells were washed to remove the extracellular bacteria, treated with gentamicin and used for the co-culture studies. The ability of the pulsed APCs to process and present the ovalbumin derived from the bacteria on MHC II molecules for T cell activation, was determined by co-culturing them with CFSE labeled OTII T cells (5×10^5^ cells/well) for a period of 4 days, as described previously [14]. At the end of the culture period, the cells were counted and stained for flow cytometric analysis.

### *In vivo* antigen presentation assay

2×10^6^ purified OT1I cells (CD45.1) were adoptively transferred (intravenously) into B6 (CD45.2) recipient mice and the mice were infected orally 24 hours later. For infections, log phase cultures of the various bacterial strains were washed and re-suspended in PBS and administered by oral gavage (10^9^ bacteria/mouse). 10 minutes before infection mice were given 0.1 ml of 5% NaHCO_3_ to neutralize the stomach acidity. The mice were sacrificed on day 7 post infection and the spleens and mesenteric lymph nodes were harvested for flow cytometric analysis.

### Flow-cytometry

Lymphocytes were isolated from spleens and cervical lymph nodes by mechanical homogenization followed by lysis of RBCs (for spleens) using lysis buffer (0.846% NH_4_Cl). Freshly isolated cells were stained with the antibodies purchased from eBioscience (San Diego, CA) or BD Biosciences (San Jose, CA). The stained samples were run on a FACSCanto (BD, San Jose, CA) and results were analyzed using FlowJo software (TreeStar Inc., Ashland, OR).

### Statistical significance

Statistical significance of differences between the various groups was tested using the student’s *t* test and p < 0.05 was considered significant.

## Competing interests

The authors declare that they have no competing interests.

## Authors’ contributions

JZ carried out all the genetic engineering. JZ and LDM carried out the protein expression studies. BJ carried out the cellular immunoassays and animal studies. DMS, WC and BJ conceived the study. DMS and BJ designed the experiments, and drafted the manuscript. All authors contributed to the preparation of the manuscript. All authors read and approved the final manuscript.
